# The acute effects of alcohol on auditory thresholds

**DOI:** 10.1186/1472-6815-7-4

**Published:** 2007-09-18

**Authors:** Tahwinder Upile, Fabian Sipaul, Waseem Jerjes, Sandeep Singh, Seyed Ahmad Reza Nouraei, Mohammed El Maaytah, Peter Andrews, John Graham, Colin Hopper, Anthony Wright

**Affiliations:** 1 Head & Neck Unit, University College London Hospitals, London, UK; 2 Royal National Throat, Nose and Ear Hospital, London, UK

## Abstract

**Background:**

There is very little knowledge about alcohol-induced hearing loss. Alcohol consumption and tolerance to loud noise is a well observed phenomenon as seen in the Western world where parties get noisier by the hour as the evening matures. This leads to increase in the referrals to the "hearing aid clinic" and the diagnosis of "cocktail party deafness" which may not necessarily be only due to presbyacusis or noise-induced hearing loss.

**Methods:**

30 healthy volunteers were recruited for this trial which took place in a controlled acoustic environment. Each of the individuals was required to consume a pre-set amount of alcohol and the hearing was tested (using full pure tone audiogram) pre- and post- alcohol consumption over a broad range of 6 frequencies. Volunteers who achieve a minimum breath alcohol threshold level of 30 u/l had to have second audiogram testing. All the volunteers underwent timed psychometric and visuo-spatial skills tests to detect the effect of alcohol on the decision-making and psychomotor co-ordination.

**Results:**

Our results showed that there was a positive association between increasing breath alcohol concentration and the magnitude of the increase in hearing threshold for most hearing frequencies. This was calculated by using the Pearson Regression Coefficient Ratio which was up to 0.6 for hearing at 1000 Hz. Over 90% of subjects had raised auditory thresholds in three or more frequencies; this was more marked in the lower frequencies.

**Conclusion:**

Alcohol specifically blunts lower frequencies affecting the mostly 1000 Hz, which is the most crucial frequency for speech discrimination. In conclusion alcohol does appear to affect auditory thresholds with some frequencies being more affected than others.

## Background

Alcohol affects every organ in the body. It is known as a central nervous system depressant and it is rapidly absorbed from the stomach and small intestine into the bloodstream. Metabolism occurs in the liver; however, the liver can only metabolize a small amount of alcohol at a time, leaving the excess to circulate throughout the body. The intensity of the effect of alcohol on the body is directly related to the amount consumed [1,2].

Individual reactions to alcohol vary, and are influenced by many factors, including but not limited to age, gender, race, physical condition, amount of food consumed before drinking, use of medications or drugs and family history of alcohol problem [1,2].

Alcohol consumption and tolerance to loud noise is a well observed phenomenon as seen in the Western world where parties get noisier by the hour as the evening matures. This leads to increase in the referrals to the "hearing aid clinic" and the diagnosis of "cocktail party deafness" which may not necessarily be only due to presbyacusis or noise-induced hearing loss.

Auditory evoked potentials and magnetic fields elicited by infrequent deviant tones differing in frequency (5% and 20% change) and novel sounds were recorded with whole-head magnetoencephalography (MEG) and electroencephalography (EEG) in eleven right-handed subjects in a double-blind, placebo-controlled (0.8 g/kg ethanol or juice), cross-over design. Kahkonen et al. concluded that alcohol impairs the processing of tones, frequency change and novel sounds at different phases of auditory processing similarly in both hemispheres [3].

In humans, acute alcohol consumption to the intoxication level may cause a temporary reduction in distortion product otoacoustic emissions amplitudes at high frequencies without affecting auditory thresholds [4]. Verma et al. [5] studied the audiovestibular function in patients of long-term alcohol dependence and compared these changes with social users of alcohol and complete abstainers. They were able to show that elevated thresholds at higher frequencies can be the only abnormality in alcohol-dependent patients.

Auditory threshold (AT) measurement method has become the standard behavioral procedure for describing auditory sensitivity. Therefore, the AT measurement method is applicable in evaluating auditory function. However, only a few studies have been performed to clarify the alteration of audibility under the influence of alcohol on normal humans by measurement of AT [6]. Murata et al. conducted a study to elucidate how alcohol ingestion method affects the auditory threshold at a wider range from lower to higher frequency in the time course. Their results showed that drinking extra small amounts of alcohol induces the elevation of AT (deterioration of hearing); they also found that the effect of alcohol on AT is altered by the alcoholic dose used [6].

We have recently studied the effect of alcohol on sound perception in cochlear implant users, finding that alcohol significantly increased the upper end of the dynamic range ('comfort level') in comparison with placebo. This effect was likely to be the result of change in the auditory pathways proximal to the cochlea [7].

The aim of this study was to determine whether alcohol could affect auditory thresholds in volunteers under controlled acoustic environment using full pure tone audiogram.

## Methods

Thirty healthy volunteers were recruited for this trial which took place in a controlled acoustic environment. The trial protocol was approved by the University of London Joint Ethics Committee. Demographical information on each volunteer included: general health, drinking and smoking habits, noise exposure and other recreational activities.

An information sheet explaining the aim of our study in simple non-scientific terms was given to each volunteer who was then asked to sign a consent form prior to the trial. Inclusion criteria were healthy volunteers over 18 years of age with no known abnormality of the hearing and balance (vestibulocochlear) systems. Volunteers were excluded from this trial if they failed to reach a minimum breath alcohol threshold level of 30 u/l and/or psychometric and visuo-spatial skills tests before the second audiogram testing. The minimum breath alcohol threshold level of 30 u/l was chosen, as this is the legal driving limit in the United Kingdom; it was felt to be better appreciated by the non-specialist.

Each of the individuals was required to consume alcohol, to give a breath alcohol concentration of 30 u/l or more, and the hearing was tested (using full pure tone audiogram) pre- and post- alcohol consumption over a broad range of 6 frequencies. All volunteers underwent timed psychometric and visuo-spatial skills tests before each hearing test to detect the effect of alcohol on the decision-making and psychomotor ability in order to be able to comply with the performance of the audiogram. The hearing test was conducted by a qualified sober audiologist in a controlled acoustic-barrier environment. Individuals with a change in their audiogram were invited back for audiometric testing the next day and over the following week.

Further audiogram testing was carried out for volunteers who achieved a minimum breath alcohol threshold level of 30 u/l and who showed satisfactory psychometric and visuo-spatial skills tests before and after alcohol ingestion

### Statistical analysis

Collected data was analysed using Graph Pad Prism 4.0 software. Wilcoxon signed rank tests for significance with p < 0.01 and Pearson Regression Coefficient Ratio were used.

## Results

**Figure 1:**
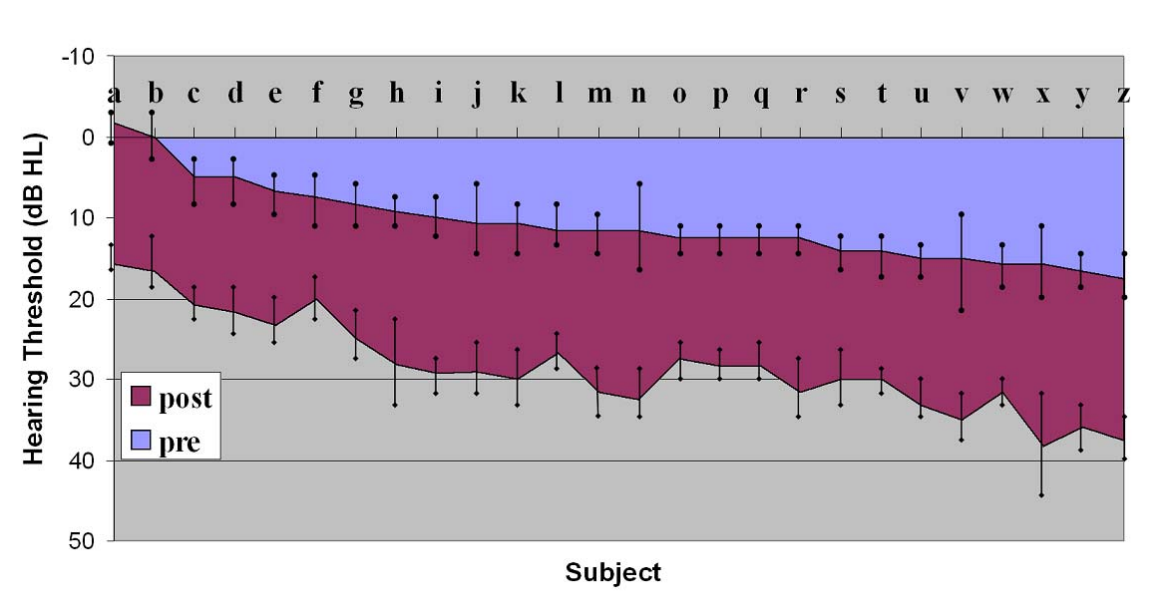
The Mean Hearing Thresholds over six frequencies (250 Hz, 500 Hz, 1000 Hz, 2000 Hz, 4000 Hz, 8000 Hz) pre- and post- alcohol consumption, to give a breath alcohol concentration of 30 u/l or more, (subjects a-z ranked). The trend for the maximum hearing threshold change (mean 15 ± 7 dB) is reflected by the lower coloured (i.e. post alcohol) area of the graph.

The mean age of the subjects tested was 27 ± 5 years (range 20–40). The mean breath alcohol concentration was 62 u/l. Four volunteers were excluded from a second hearing test because of failure of competence testing. Alcohol increased the hearing threshold in all individuals, affecting some frequencies more than others, making it more difficult to correctly perceive a given pure tone, with a mean change of 7dB with 90% of subjects having 3 or more frequencies affected; figure 1 shows the mean hearing thresholds over six frequencies (250 Hz, 500 Hz, 1000 Hz, 2000 Hz, 4000 Hz, 8000 Hz) pre- and post- alcohol consumption (Table 1). Wilcoxon signed rank testing showed a significant difference (P < 0.0001) between pre and post alcohol auditory thresholds.

**Table 1:** The Mean hearing loss in decibels for each frequency tested

Sound Frequency (Hz)	Mean loss male n = 11 (dB)	Mean loss female n = 15 (dB)
250	6	12
500	5	17
1000	3	10
2000	2	5
4000	5	7
8000	9	8

There was a positive association between increasing breath alcohol concentration and the magnitude of the increase in hearing threshold for most hearing frequencies. This was calculated by using the Pearson Regression Coefficient Ratio, which was up to 0.6 for hearing at 1000 Hz. Over 90% of subjects had three or more frequencies affected (mean of 5 frequencies affected ± 1.0); this is more marked in the lower frequencies (Table 1). The mean value for the maximum hearing threshold change in the worst affected frequency was 15 ± 7 dB for the population tested. Subgroup analysis suggested that the hearing thresholds of women in our study were more affected than men; this was more marked, again in the lower frequencies. Slim and healthy people were least affected, whilst older subjects or those with a previous history of heavy drinking were most affected.

The affects of alcohol on raising hearing thresholds appear reversible having retested some individuals over the week after the trial, by which time the audiograms tend to return to pre-alcohol intake levels. Some individuals also experienced transient tinnitus that could be related to alcohol intake.

## Discussion

Social drinking in the evening usually augments the noise-induced hearing loss people sustain during working hours in the modern industrial world. The hearing loss that is seen during the aging process is often attributed to presbyacusis and noise exposure but alcohol may also play a significant role in hearing loss (alcohol-induced hearing loss). The popular "hearing clinic" referral diagnosis of "cocktail party deafness" cannot necessarily be attributed to the aging process alone or noise-induced hearing loss. Alcohol consumption is shown to cause a temporary threshold shift in already aging hearing mechanism, and possibly over time these changes may become permanent [1-3].

Acoustic reflex thresholds were measured for eighteen young adults (9 men and 9 women) at four different blood alcohol levels: 0.00%, ascending 0.10%, 0.15% (peak level), and descending 0.10%. Reflex-eliciting stimuli consisted of three narrow-band noises (300 to 600, 600 to 1200, and 1200 to 2400 Hz) and three broad band noises (white noise, recorded rock music, and recorded factory noise). Pre-alcohol reflex thresholds were found to be significantly more sensitive than all post-alcohol reflex thresholds for all stimuli, and broad-band stimuli demonstrated greater threshold shifts than did narrow-band stimuli. Significant sex differences were not observed for any blood alcohol level [8].

Popelka et al. conducted a study using lower levels of alcohol over only two frequencies in five subjects with normal hearing and found a reduction in hearing ability. Specifically, acoustic reflex thresholds were raised, reflex magnitude decreased, and temporary threshold shift increased under alcohol conditions [9]. An earlier cross-sectional study, although not specifically looking at alcohol consumption and hearing, showed a frequency-specific effect in which low frequencies were more severely affected than higher ones. This contradicts our finding that the effect of alcohol on hearing varies with degree of exposure and gender. However, the investigators did find an increase in the probability of having a hearing loss over the high frequencies in those with a history of heavy drinking [10].

Our results have shown a frequency-specific effect in which low frequencies were more severely affected than higher ones. This frequency-specific effect was confirmed by other studies [9-11]; although they found an increase in thresholds at the frequencies important for speech discrimination above 1000 Hz which was nearly three times greater than that for lower frequencies. This difference is probably attributed to the fact that we examined a much younger cohort of individuals with little pre-existing hearing pathology.

The results are corroborated by our recent Cochlear Implantation study on the effect of alcohol on loudness discomfort levels (as a proxy measure of auditory function) whereby alcohol raised the threshold of perception of discomfort in the residual cochlear of Cochlear Implantation patients [7].

Our study suggests that alcohol preferentially blunts the lower frequencies thresholds including 1000 Hertz, which is the most important frequency to discriminate vowels. The reduction in hearing in these frequencies is more detrimental to understanding of the human speech. The mild to moderate consumption of alcohol affects the hearing thresholds to dull the pure tones in speech frequencies. We feel that if the hearing had been assessed by speech audiometry, the disability would have been more since alcohol is also known to act at a cortical level causing significant deterioration in speech discrimination. Alcohol may act peripherally by a direct toxic or osmotic effect or more centrally disrupting processing of auditory information [10,11]. Alcohol consumption in moderate amounts has been shown to alter the central auditory processing under difficult listening conditions [11]. Investigators have suggested the hypothesis that alcohol acts centrally, at the level of mechanisms involved in the temporal and binaural summation of auditory signals, rather than influencing peripheral structures [11,12]. The effect of alcohol on hearing was also found to be reversible in the short term [11] but long-term permanent threshold changes cannot be excluded.

## Conclusion

The limited power of this study precludes stringent subgroup analysis. A more formal study with a greater numbers of participants and measurement of both blood and breath alcohol levels would by no doubt lead to the increased accuracy and scientific validation of results. Pure tone and speech audiometry, perhaps supplemented with stapedial reflex changes or evoked response audiometry, may help to further elucidate the actual hearing pathways, central and/or peripheral affected by alcohol. There remains a huge scope for further research.

## Competing interests

The author(s) declare that they have no competing interests.

## Authors' contributions

**TU**: designed the study, carried out the literature research, clinical study, manuscript preparation and manuscript review. **SF**: carried out the literature research, clinical study and manuscript preparation and manuscript review. **WJ**: carried out the literature research, clinical study and manuscript preparation and manuscript review. **SS: **carried out the literature research, clinical study and manuscript preparation and manuscript review. **SN: **contributed to conception and design, carried out the manuscript editing and manuscript review. **ME: **carried out the literature research, clinical study and manuscript preparation and manuscript review. **AP**: contributed to conception and design, carried out the manuscript editing and manuscript review. **GJ**: contributed to conception and design, carried out the manuscript editing and manuscript review. **CH**: contributed to conception and design, carried out the manuscript editing and manuscript review. **AW**: designed the study, carried out the literature research, clinical study and manuscript preparation.

All authors read and approved the final manuscript.

## Pre-publication history

The pre-publication history for this paper can be accessed here:


